# Increased digital media use is associated with sleep problems among university students: A study during the COVID-19 pandemic in Japan

**DOI:** 10.3389/fpsyt.2022.946265

**Published:** 2022-08-29

**Authors:** Kasumi Watanabe, Hiroyoshi Adachi, Ryohei Yamamoto, Ryohei Fujino, Daiki Ishimaru, Daisuke Kanayama, Yukako Sakagami, Shoshin Akamine, Noriko Marutani, Yoshimasa Mamiya, Midori Mashita, Natsuko Nakano, Takashi Kudo, Manabu Ikeda

**Affiliations:** ^1^Department of Psychiatry, Osaka University Graduate School of Medicine, Osaka, Japan; ^2^Health and Counseling Center, Osaka University, Osaka, Japan; ^3^Osaka University Hospital, Sleep Medicine Center, Osaka, Japan; ^4^Graduate School of Human Sciences, Osaka University, Osaka, Japan

**Keywords:** digital media use, university students, COVID-19, retrospective study, sleep problems

## Abstract

This retrospective cohort study investigates the association between the incidence of sleep problems and changes in digital media use among university students during the COVID-19 pandemic. It used data from annual health check-ups performed at a Japanese university in 2019 and 2020. Students undergoing these check-ups were identified to respond to questions about sleep problems, digital media use, breakfast and exercise habits, and stress. In total, 3,869 students were included in the analysis. The association between the incidence of sleep problems in 2020 and the changes in digital media use between 2019 and 2020 was assessed using logistic regression models. The rate of long digital media use (≥ 2 hours) in 2019 was 42.6%, while in 2020 it was 53.6%. Incidence of sleep problems was observed in 244 students (6.3%) in 2020. There were 786 students (20.3%) who used digital media for ≤ 2 h in 2019 and ≥ 2 h in 2020. From the sample, 66 students (8.4%) reported incidence of sleep problems in 2020. Additionally, those respondents who specifically reported increased digital media use between 2019 and 2020 (increased use) where at greater risk (OR: 1.76; 95% CI: 1.21, 2.55) of reporting sleep problems in 2020, even after controlling for other study variables. Thus, this study provides evidence that the incidence of sleep problems has had a significant association with an increase in digital media use among university students throughout the COVID-19 pandemic. These findings highlight the importance of ensuring appropriate digital media use among students for improved quality of sleep.

## Introduction

Since 2020, the world has been witnessing the spread of the severe acute respiratory syndrome coronavirus 2 (SARS-CoV-2), which has resulted in the COVID-19 pandemic. In Japan, the first case of COVID-19 infection was reported on 15 January 2020, and the Japanese government declared a state of emergency on 7 April 2020. To prevent the spread of infection, educational institutions were required to discontinue in-person teaching, and to conduct classes and share learning material online. Most students had to stay home and study by themselves, without face-to-face communication with teachers and friends. Such isolation resulted in severe psychological stress among university students (1, 2).

Studies have reported that increased levels of psychological distress during the COVID-19 pandemic were associated with sleep problems (3). The unusual conditions associated with the pandemic affected students' lifestyles, including sleep habits. A recent study in China revealed increased screen and sleep time, and decreased physical activity, among adolescents (4). Studies in Italy revealed delayed sleep rhythms and poor sleep quality during the COVID-19 lockdown (5, 6). In Spain, nursing students spent more time in bed during the lockdown but displayed an adverse quality of sleep (7).

Reports have indicated that insomnia may be associated with stressful environments (8, 9). A study from China revealed that the prevalence of insomnia symptoms in college students during the COVID-19 pandemic was 25.7% (10). This figure exceeds the 23.6% prevalence reported for Chinese university students during the pre-COVID-19 period (11).

Extensive social media use among students is associated with poorer sleep patterns, delayed onset of sleep, delayed waking up time on school days, and difficulty falling asleep after waking up (12). During the pandemic, many university students in home quarantine were exposed to stressful COVID-19-related information through social media (13); thus, an increased fear of COVID-19 might have partially affected the rise in sleep problems (14). Another cross-sectional study reported that addiction to social media during the COVID-19 pandemic was positively associated with poor sleep, anxiety, and depression in Bangladeshi college and university students (15).

Previous research has described several possible mechanisms for how digital media use might impact sleep. First, time spent using digital media might directly replace sleep time or other habits related to better sleep. Second, emotional stimulation caused by digital media might cause sleep problems *via* physiological reactions (16, 17). Third, exposure to blue light might affect physiological functions, including circadian rhythms (18). Light-emitting diodes, which are used in computers and smartphones, have the strongest intensity in blue wavelengths (400–490 nm) of the visible light range (18). Chang and colleagues (19) established that the use of light-emitting electronic devices before bedtime inhibits melatonin secretion and disrupts circadian rhythms. Melatonin is a hormone produced in the corpus at night and plays an important role in sleep and mood management synchronizing circadian rhythms (20). The abovementioned reports indicate that exposure to blue light from electronic devices can negatively affect sleep patterns by inhibiting the melatonin pathway.

This study investigates the association between the incidence of sleep problems in 2020 and changes in digital media use from 2019 to 2020 among university students in Japan. To the best of our knowledge, no cohort study has reported an association between the frequency of sleep problems and increased digital media use among university students during the COVID-19 pandemic. We believe that the findings of this retrospective cohort study will encourage university students to be more cautious about their digital media use and improve their sleep quality.

## Materials and methods

### Study participants and procedure

**Figure 1:**
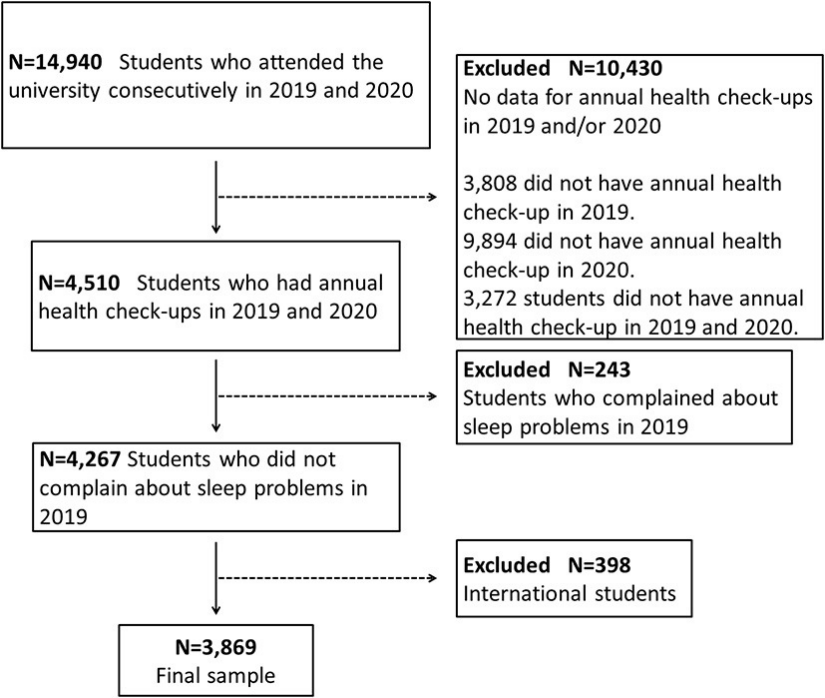
Flow diagram of participant selection.

Of the 14,940 students who attended Osaka University in 2019 and 2020, 4,510 students who underwent annual health check-ups in April were eligible for this study (response rate: 30.2%). The students accessed the website for annual health check-ups operated by Osaka University and answered an online questionnaire about sleep problems, digital media use, stress, and their habits at the time of response. The questionnaire was answered in April of each fiscal year. We excluded the following individuals: (1) students who reported having sleep problems in 2019 (*n* = 243; 5.4%); and (2) international students (*n* = 398; 8.8%). The final sample included 3,869 students (85.8% of those initially selected; see Figure 1). This study used an opt-out approach for informed consent, according to the Japanese Ethical Guidelines for Medical and Health Research Involving Human Subjects. According to the Osaka University and government regulations, all students were required to undergo annual health check-ups. Students were informed in the privacy policy that the collected data would be anonymized and used for future research and operational improvements. The protocol for this study was approved by the Ethics Committee of the Health and Counseling Center, Osaka University (number 14, 2022) and the Ethics Committee of the Osaka University Hospital (number 18352-2, 2022). The details of the study were presented on the university's website, and a contact point was set up to ensure that participants could opt out of the use of existing data. All data was retrieved from the electronic database of the Health and Counseling Center, Osaka University. All procedures in this study involving human participants were performed in accordance with the ethical standards of the institutional and national research committee and with the 1964 Helsinki Declaration and its later amendments or comparable ethical standards.

### Measures

The students were required to answer questions at each health check-up in 2019 and 2020, as described below:

#### Sleep problems

Sleep problems were assessed based on the following question: “Do you have difficulty in sleeping?” Two response options were provided: “yes” and “no”.

#### Digital media use

Digital media use was determined by the following question: “How many hours a day do you spend using a social networking service and email, surfing the Internet, or playing games?” It was accompanied by five response options: “30 min or less,” “30–60 min,” “1–2 h,” “2–3 h,” and “3 h or more.” In this study, short digital media use was defined as ≤ 2 h and long digital media use as ≥ 2 h based on the study by Wu et al. (21). We set the cut-off point at 2 h; the students using digital media for ≥ 2 h were assumed to be at high risk of sleep problems. To assess changes in digital media use, the students were divided into four groups: Short-Short, Short-Long, Long-Short, and Long-Long, based on how much time they spent using digital media in 2019 and 2020. For example, the “Short-Long” group included students who used digital media for ≤ 2 h in 2019 and ≥ 2 h in 2020.

#### Stress

To assess how often they felt stressed, students were asked, “Do you feel stressed?” and instructed to respond by choosing one of the following options: “almost never,” “sometimes,” “often,” and “always”.

#### Breakfast habits

To assess students' eating habits, the question, “Do you eat breakfast?” was posed and accompanied by four response options, namely: “almost always,” “usually,” “sometimes,” and “almost never”.

#### Exercise habits

To assess students' exercise habits, students were asked, “How many days a week do you perform exercises?” and offered five response choices: “0 days,” “1 day,” “2 days,” “3 or 4 days,” and “5 or more days”.

### Statistical analyses

Digital media use was used to classify students' demographic characteristics. The chi-square test and one-way ANOVA were used to assess differences in characteristics among the four groups classified by digital media use.

Univariable and multivariable logistic regression models using the incidence of sleep problems in 2020 as a dependent variable were used to calculate unadjusted and adjusted odds ratios (OR) with 95% confidence intervals (CI) of age, sex, exercise and breakfast habits in 2020, and the change in digital media use.

Continuous variables were expressed as mean ± standard deviation (SD) and categorical variables were expressed as numbers and proportions. Statistical significance was set at *P* < 0.05. All statistical analyses were performed using SPSS version 26 (SPSS Inc., Chicago, IL, USA).

## Results

### Characteristics of the participants in 2020 by changes in digital media use from 2019 to 2020

Table 1 shows the characteristics of the 3,869 students according to the changes in digital media use from 2019 to 2020. The sample comprised 2,484 male (64.2%) and 1,385 female (35.8%) students, with a mean age of 22.7 years (SD = 4.0). The group that reported short digital media use in both 2019 and 2020 (Short-Short group) included 1,435 students (37.1%), whereas 786 students (20.3%) reported using digital media for more prolonged durations in 2020 than in 2019 (Short-Long group). A total of 1,287 students (33.3%) reported long durations of digital media use in both 2019 and 2020. The rate of long digital media use in 2019 was 42.6%, while in 2020 it was 53.6%. Incidence of sleep problems was observed in 244 students (6.3%) in 2020. In the Short-Long group, 66 students (8.4%) reported incidence of sleep problems, which was higher than that in all the other groups. Regarding the degree of stress, almost half of the students in each group reported feeling stressed “sometimes.” Regarding the habit of eating breakfast, more than half of the students in each group responded with “almost always.” Moreover, over 40% of the students reported no exercise habits.

**Table 1:** Students' characteristics in 2020 according to changes in digital media use from 2019 to 2020.

		**Total**		**Short-Short**		**Short-Long**		**Long-Short**		**Long-Long**		***p-value***
		***n** =* **3,869**		***n** =* **1,435**		***n** =* **786**		***n** =* **361**		***n** =* **1,287**		
Age, years^a^		22.7 ± 4.0		23.8 ± 5.5		22.2 ± 2.5		22.3± 3.0		22.0 ± 2.5		<0.001
Male, *n* (%)		2,484	(64.2%)	912	(63.6%)	501	(63.7%)	210	(58.2%)	861	(66.9%)	0.018
Female, *n* (%)		1,385	(35.8%)	523	(36.4%)	285	(36.3%)	151	(41.8%)	426	(33.1%)	
Incidence of sleep problems, *n* (%)		244	(6.3%)	61	(4.3%)	66	(8.4%)	26	(7.2%)	91	(7.1%)	<0.001
Stress, *n* (%)	Almost never	1,299	(33.6%)	521	(36.3%)	247	(31.4%)	118	(32.7%)	413	(32.1%)	0.214
	Sometimes	1,981	(51.2%)	716	(49.9%)	417	(53.1%)	189	(52.4%)	659	(51.2%)	
	Often	495	(12.8%)	168	(11.7%)	102	(13.0%)	42	(11.6%)	183	(14.2%)	
	Always	94	(2.4%)	30	(2.1%)	20	(2.5%)	12	(3.3%)	32	(2.5%)	
Breakfast, *n* (%)	Almost always	2,298	(59.4%)	929	(64.7%)	445	(56.6%)	228	(63.2%)	696	(54.1%)	<0.001
	Usually	720	(18.6%)	265	(18.5%)	147	(18.7%)	63	(17.5%)	245	(19.0%)	
	Sometimes	513	(13.3%)	138	(9.6%)	124	(15.8%)	36	(10.0%)	215	(16.7%)	
	Almost never	338	(8.7%)	103	(7.2%)	70	(8.9%)	34	(9.4%)	131	(10.2%)	
Exercise, *n* (%)	0 days per week	1,791	(46.3%)	626	(43.6%)	351	(44.7%)	162	(44.9%)	652	(50.7%)	0.046
	1 day per week	760	(19.6%)	301	(21.0%)	152	(19.3%)	69	(19.1%)	238	(18.5%)	
	2 days per week	580	(15.0%)	237	(16.5%)	116	(14.8%)	52	(14.4%)	175	(13.6%)	
	3 to 4 days per week	514	(13.3%)	184	(12.8%)	122	(15.5%)	55	(15.2%)	153	(11.9%)	
	5 or more days per week	224	(5.8%)	87	(6.1%)	45	(5.7%)	23	(6.4%)	69	(5.4%)	

^a^Mean ± standard deviation.

### The association between the incidence of sleep problems and changes in digital media use from 2019 to 2020

As shown in Table 2, the Short-Long group in digital media use was significantly associated with the incidence of sleep problems in the unadjusted model (OR: 2.07; 95% CI: 1.44, 2.96), and even after adjusting for all other factors (adjusted OR: 1.76; 95% CI: 1.21, 2.55). Frequency of stress was significantly associated with the incidence of sleep problems, and students with higher stress tended to show higher odds of sleep problems. As for breakfast, “Usually” and “Sometimes” were significantly associated with the incidence of sleep problems. However, no significant association was found between frequency of exercise and the incidence of sleep problems.

**Table 2:** The association between the incidence of sleep problems and changes in digital media use from 2019 to 2020.

		**Unadjusted model**		**Adjusted model**	
		**OR [95%CI]**	***p-value***	**OR [95%CI]**	***p-value***
Age, per 1 year		0.95 [0.91, 1.00]	0.026	0.93 [0.88, 0.98]	0.006
Female (vs. Male)		0.94 [0.71, 1.23]	0.644	0.85 [0.64, 1.14]	0.274
Stress	Almost never	0.52 [0.36, 0.74]	<0.001	0.51 [0.35, 0.73]	<0.001
	Sometimes	1.00 [reference]		1.00 [reference]	
	Often	2.06 [1.47, 2,87]	<0.001	2.13 [1.52, 2.99]	<0.001
	Always	6.70 [4.15, 10.82]	<0.001	7.66 [4.63, 12.66]	<0.001
Digital media use	Short-Short	1.00 [reference]		1.00 [reference]	
	Short-Long	2.07 [1.44, 2.96]	<0.001	1.76 [1.21, 2.55]	0.003
	Long-Short	1.75 [1.09, 2.81]	0.021	1.53 [0.94, 2.50]	0.088
	Long-Long	1.71 [1.23, 2.39]	0.002	1.40 [0.99, 1.98]	0.057
Breakfast	Almost always	1.00 [reference]		1.00 [reference]	
	Usually	1.53 [1.09, 2.13]	0.013	1.44 [1.02, 2.03]	0.037
	Sometimes	1.90 [1.33, 2.70]	<0.001	1.66 [1.14, 2.41]	0.008
	Almost never	1.63 [1.06, 2.52]	0.027	1.44 [0.91, 2.27]	0.119
Exercise	0 days per week	1.00 [reference]		1.00 [reference]	
	1 day per week	0.74 [0.51, 1.08]	0.115	0.88 [0.60, 1.30]	0.519
	2 days per week	1.13 [0.78, 1.62]	0.523	1.34 [0.92, 1.95]	0.126
	3 to 4 days per week	0.93 [0.62, 1.40]	0.735	1.05 [0.69, 1.60]	0.806
	5 or more days per week	0.80 [0.43, 1.47]	0.462	0.88 [0.47, 1.66]	0.695

OR, odds ratio; CI, confidence interval.

## Discussion

This study investigated the association between the incidence of sleep problems and changes in digital media use from 2019 to 2020 among university students during the COVID-19 pandemic. Incidence of sleep problems was observed in 244 students (6.3%) in 2020. We assessed additional factors that may affect students' sleep patterns. Both breakfast and exercise habits are considered important lifestyle factors that affect sleep (22, 23). During the COVID-19 pandemic, a lack of exercise habits was reported as a risk factor for sleep disorders in China (24). Therefore, in addition to digital media use, these habits were assessed. Furthermore, the degree of stress, which is considered to be a significant risk factor for sleep problems experienced by students, was assessed. Even after adjusting for the abovementioned factors, an increase in digital media use from 2019 to 2020 was significantly associated with the incidence of sleep problems in 2020.

In the adjusted model for other risk factors of sleep problems, the Short-Long group had a change in digital media exposure to ≥ 2 h, which may have caused sleep problems through some physiological responses. In contrast, the Long-Short group did not show a significant risk of sleep problems caused by digital media use. These results imply that digital media use of ≤ 2 h might not increase the risk of sleep problems compared to that of ≥ 2 h. The Long-Long group showed lower odds of sleep problems than the Short-Long group. That could possibly be because students who use digital media of ≥ 2 h already had sleep problems in 2019 and might have been excluded during participant selection. With respect to the Long-Long group, no statistically significant association was found, but there was a tendency toward increased risk with a *p*-value of 0.057. Thus, it is considered that this does not rule out an association between digital media use of ≥ 2 h over a long period and the risk of sleep problems.

A recent systematic review and meta-analysis showed that the prevalence of insomnia among populations affected by COVID-19 was 23.9% (25). To our knowledge, no study has longitudinally investigated the incidence of sleep problems among university students during the COVID-19 pandemic. However, Carter and colleagues revealed that bedtime media device use was strongly associated with poor sleep quality and daytime sleepiness (26). In particular, the COVID-19 pandemic has resulted in an increased reliance on various electronic devices to connect to the Internet, leading to increased screen time (27). Furthermore, a cross-sectional study in Bangladesh found that addiction to social media was positively associated with poor sleep among college and university students (15). In this study, the incidence of sleep problems was significantly associated with an increase in digital media use from ≤ 2 h to ≥ 2 h. Wu et al. (21) showed that high screen time, categorized as >2 h per day, was significantly positively correlated with poor sleep quality among Chinese college students. The results of this study are consistent with the abovementioned finding.

This study has some limitations. First, the participants were all students of the same university. The effects of the COVID-19 pandemic might vary depending on where the students live. It has been reported that living in cities is a risk factor for insomnia symptoms, compared with living in rural areas (10). In addition, the lower the level of knowledge about COVID-19, the higher the prevalence of insomnia symptoms among students (10). This result implies that providing relevant and accurate COVID-19-related information might protect university students from sleep problems. Therefore, it is necessary to verify whether the present results are reproducible in future studies in other universities. Second, this study was based on students' subjective reports during annual health check-ups. In particular, “sleep problems” were not based on a clinical diagnosis as represented by the DSM-5. During the check-ups, sleep problems were assessed by one yes-or-no question rather than the degree of sleep problems. Therefore, this study could not assess changes in sleep problem severity. Thus, future studies using a structured measurement tool, such as the Pittsburgh Sleep Quality Index, are required to assess sleep problems and their components in detail. Further research is also needed to objectively measure sleep parameters using other methods, such as actigraphy. Third, the degree of stress was defined by only one question for the reasons mentioned above. The use of a comprehensive stress-assessment questionnaire is warranted in further studies to examine the impact of stress on our results in more detail. Fourth, timeframes were not set for each question at the health check-ups. The students reported their sleep problems, digital media use, stress, and habits at the time of response. The effect of independent factors on the incidence of sleep problems might vary depending on the duration of exposure to each factor. Fifth, breakfast and exercise habits were assessed by a question about frequency. In this study, no stepwise association in breakfast habits was found such that students who ate breakfast less frequently were related with incidence of sleep problems. Regarding exercise, there was no significant association between the frequency of exercise and incidence of sleep problems. In addition to frequency of breakfast or exercise, contents might influence the incidence of sleep problems (23, 28), however, this study could not assess these aspects in detail. Sixth, the sample in this study included students who had undergone annual health check-ups in two consecutive years (2019 and 2020), as described in the participant selection flow diagram. Of the 14,940 students, 3,808 (dropout rate: 25.5%) did not have a health check-up in 2019 and 9,894 (dropout rate: 66.2%) did not have one in 2020. The dropout rate in 2020 was very high, which might be due to limitations on receiving health check-ups during the COVID-19 pandemic. Such a high exclusion rate in the target population could be considered a selection bias in the study sample. Finally, this study could not assess the sleep problems of international students. International students were excluded during participant selection because they tend to contact their family and friends in their home country while adjusting to different time zones, which might make differences in the time using digital media. Additionally, international students who went home for spring vacation and could not return to Japan due to the COVID-19 pandemic might answer the questionnaire at health check-ups from their home country in 2020. International students were considered unsuitable for the purpose of this study because of differences in their lifestyles and surroundings.

## Conclusions

This study assessed the relationship between the incidence of sleep problems and changes in digital media use among university students during the COVID-19 pandemic. The results showed that incidence of sleep problems was significantly associated with an increase in digital media use, even after adjusting for the effects of related and underlying factors. This is the first study that investigated the relationship between incidence of sleep problems and changes in digital media use during the COVID-19 pandemic among university students in Japan. The results provide a warning to university students regarding long digital media use, and highlight the need to ensure appropriate digital media use to maintain sleep quality, particularly during situations that can result in abrupt lifestyle changes.

## Data availability statement

The data analyzed in this study is subject to the following licenses/restrictions: The datasets generated during the current study are not publicly available because of privacy considerations regarding the participants. Requests to access these datasets should be directed to HA, hadachi@psy.med.osaka-u.ac.jp.

## Ethics statement

The studies involving human participants were reviewed and approved by the Ethics Committee of the Health and Counseling Center, Osaka University (number 14, 2022) and Ethics Committee of the Osaka University Hospital (number 18352-2, 2022). The Ethics Committee waived the requirement of written informed consent for participation.

## Author contributions

Conceptualization and formal analysis: KW and HA. Methodology: KW, HA, and RY. Investigation and writing—original draft draft preparation: KW. Data curation: RY. writing—review and editing: HA, RY, RF, DI, DK, YS, SA, NM, YM, MM, NN, TK, and MI. All authors contributed to writing the final manuscript and approved the final version.

## Funding

This work was supported by the Innovation Platform for Society 5.0 from the Japan Ministry of Education, Culture, Sports, Science and Technology (Code: S004541) and Japan Society for the Promotion of Science (JSPS) KAKENHI (Grant Number 22K03123).

## Conflict of interest

The authors declare that the research was conducted in the absence of any commercial or financial relationships that could be construed as a potential conflict of interest.

## Publisher's note

All claims expressed in this article are solely those of the authors and do not necessarily represent those of their affiliated organizations, or those of the publisher, the editors and the reviewers. Any product that may be evaluated in this article, or claim that may be made by its manufacturer, is not guaranteed or endorsed by the publisher.

## References

[1] Sahu P. Closure of universities due to coronavirus disease 2019 (COVID-19): impact on education and mental health of students and academic staff. Cureus. (2020) 12:e7541. 10.7759/cureus.7541 32377489 PMC7198094

[2] Arima M Takamiya Y Furuta A Siriratsivawong K Tsuchiya S Izumi M. Factors associated with the mental health status of medical students during the COVID-19 pandemic: a cross-sectional study in Japan. BMJ. (2020) 10:e043728. 10.1136/bmjopen-2020-043728 33303472 PMC7733210

[3] Alimoradi Z Brostrom A Tsang HWH Griffiths MD Haghayegh S Ohayon MM. Sleep problems during COVID-19 pandemic and its association to psychological distress: a systematic review and meta-analysis. E Clin Med. (2021) 36:e100916. 10.1016/j.eclinm.2021.100916 34131640 PMC8192091

[4] Yang S Guo B Ao L Yang C Zhang L Zhou J. Obesity and activity patterns before and during COVID-19 lockdown among youths in China. Clin Obes. (2020) 10:e12416. 10.1111/cob.12416 33009706 PMC7646045

[5] Cellini N Canale N Mioni G Costa S. Changes in sleep pattern, sense of time and digital media use during COVID-19 lockdown in Italy. J Sleep Res. (2020) 29:e13074. 10.1111/jsr.13074 32410272 PMC7235482

[6] Marelli S Castelnuovo A Somma A Castronovo V Mombelli S Bottoni D. Impact of COVID-19 lockdown on sleep quality in university students and administration staff. J Neurol. (2021) 268:8–15. 10.1007/s00415-020-10056-6 32654065 PMC7353829

[7] Romero-Blanco C Rodríguez-Almagro J Onieva-Zafra MD Parra-Fernandez ML Prado-Laguna MDL Hernandez-Martinez A. Sleep pattern changes in nursing students during the COVID-19 lockdown. Int J Environ Res Public Health. (2020) 17:5222. 10.3390/ijerph17145222 32698343 PMC7400502

[8] Nunn CL Samson DR Krystal AD. Shining evolutionary light on human sleep and sleep disorders. Evol Med Public Health. (2016) 2016:227–43. 10.1093/emph/eow018 27470330 PMC4972941

[9] Kalmbach DA Anderson JR Drake CL. The impact of stress on sleep: pathogenic sleep reactivity as a vulnerability to insomnia and circadian disorders. J Sleep Res. (2018) 27:e12710. 10.1111/jsr.12710 29797753 PMC7045300

[10] Zhou S-J Wang L-L Yang R Zhang L-G Guo Z-C Chen J-C. Sleep problems among Chinese adolescents and young adults during the coronavirus-2019 pandemic. Sleep Med. (2020) 74:39–47. 10.1016/j.sleep.2020.06.001 32836185 PMC7274988

[11] Li L Wang YY Wang S-B Zhang L Li L Xu D-D. Prevalence of sleep disturbances in Chinese university students: a comprehensive meta-analysis. J Sleep Res. (2018) 27:e12648. 10.1111/jsr.12648 29383787

[12] Scott H Biello SM Woods HC. Social media use and adolescent sleep patterns: cross-sectional findings from the UK millennium cohort study. BMJ. (2019) 9:e031161. 10.1136/bmjopen-2019-031161 31641035 PMC6830469

[13] Tang W Hu T Hu B Jin C Wang G Xie C. Prevalence and correlates of PTSD and depressive symptoms one month after the outbreak of the COVID-19 epidemic in a sample of home-quarantined Chinese university students. J Affect Disord. (2020) 274:1–7. 10.1016/j.jad.2020.05.009 32405111 PMC7217769

[14] Lin C-Y Broström A Griffiths MD Pakpour AH. Investigating mediated effects of fear of COVID-19 and COVID-19 misunderstanding in the association between problematic social media use, psychological distress, and insomnia. Internet Interv. (2020) 21:100345. 10.1016/j.invent.2020.100345 32868992 PMC7449889

[15] Islam S Sujan SH Tasnim R Mohona RA Ferdous MZ Kamruzzaman S. Problematic smartphone and social media use among Bangladeshi college and university students amid COVID-19: the role of psychological well-being and pandemic related factors. Front Psychiatry. (2021) 12:e647386. 10.3389/fpsyt.2021.647386 33935834 PMC8085355

[16] Gregory AM Sadeh A. Annual Research Review: sleep problems in childhood psychiatric disorders – a review of the latest science. J Child Psychol Psychiatry. (2016) 57:296–317. 10.1111/jcpp.12469 26412255

[17] Cain N Gradisar M. Electronic media use and sleep in school-aged children and adolescents: a review. Sleep Med. (2010) 11:735–42. 10.1016/j.sleep.2010.02.006 20673649

[18] Tosini G Ferguson I Tsubota K. Effects of blue light on the circadian system and eye physiology. Mol Vis. (2016) 22:61–72. 26900325 PMC4734149

[19] Chang A-M Aeschbach D Duffy JF Czeisler CA. Evening use of light-emitting eReaders negatively affects sleep, circadian timing, and next-morning alertness. Proc Natl Acad Sci U S A. (2015) 112:1232–7. 10.1073/pnas.1418490112 25535358 PMC4313820

[20] Rondanelli M Faliva MA Perna S Antoniello N. Update on the role of melatonin in the prevention of cancer tumorigenesis and in the management of cancer correlates, such as sleep-wake and mood disturbances: review and remarks. Aging Clin Exp Res. (2013) 25:499–510. 10.1007/s40520-013-0118-6 24046037 PMC3788186

[21] Wu X Tao S Zhang Y Zhang S Tao F. Low physical activity and high screen time can increase the risks of mental health problems and poor sleep quality among Chinese college students. PLoS ONE. (2015) 10:e0119607. 10.1371/journal.pone.0119607 25786030 PMC4364939

[22] St-Onge M-P Mikic A Pietrolungo CE. Effects of diet on sleep quality. Adv Nutr. (2016) 7:938–49. 10.3945/an.116.012336 27633109 PMC5015038

[23] Kredlow MA Capozzoli MC Hearon BA Calkins AW Otto MW. The effects of physical activity on sleep: a meta-analytic review. J Behav Med. (2015) 38:427–49. 10.1007/s10865-015-9617-6 25596964

[24] Fu W Wang C Zou L Guo Y Lu Z Yan S. Psychological health, sleep quality, and coping styles to stress facing the COVID-19 in Wuhan, China. Transl Psychiatry. (2020) 10:225. 10.1038/s41398-020-00913-3 32647160 PMC7347261

[25] Cénat JM Blais-Rochette C Kokou-Kpolou CK Noorishad P-G Mukunzi JN Mclntee S-E. Prevalence of symptoms of depression, anxiety, insomnia, posttraumatic stress disorder, and psychological distress among populations affected by the COVID-19 pandemic: a systematic review and meta-analysis. Psychiatry Res. (2021) 295:113599. 10.1016/j.psychres.2020.113599 33285346 PMC7689353

[26] Carter B Rees P Hale L Bhattacharjee D Paradkar MS. Association between portable screen-based media device access or use and sleep outcomes: a systematic review and meta-analysis. JAMA Pediatr. (2016) 170:1202–8. 10.1001/jamapediatrics.2016.2341 27802500 PMC5380441

[27] Majumdar P Biswas A Sahu S. COVID-19 pandemic and lockdown: cause of sleep disruption, depression, somatic pain, and increased screen exposure of office workers and students of India. Chronobiol Int. (2020) 37:1191–200. 10.1080/07420528.2020.1786107 32660352

[28] Tanaka E Yatsuya H Uemura M Murata C Otsuka R Toyoshima H. Associations of protein, fat, and carbohydrate intakes with insomnia symptoms among middle-aged Japanese workers. J Epidemiol. (2013) 23:132–8. 10.2188/jea.JE20120101 23419282 PMC3700250

